# What Do Transitive Inference and Class Inclusion Have in Common? Categorical (Co)Products and Cognitive Development

**DOI:** 10.1371/journal.pcbi.1000599

**Published:** 2009-12-11

**Authors:** Steven Phillips, William H. Wilson, Graeme S. Halford

**Affiliations:** 1 Neuroscience Research Institute, National Institute of Advanced Industrial Science and Technology (AIST), Tsukuba, Ibaraki, Japan; 2 School of Computer Science and Engineering, The University of New South Wales, Sydney, New South Wales, Australia; 3 School of Psychology, Griffith University, Brisbane, Queensland, Australia; John Radcliffe Hospital, United Kingdom

## Abstract

Transitive inference, class inclusion and a variety of other inferential abilities have strikingly similar developmental profiles—all are acquired around the age of five. Yet, little is known about the reasons for this correspondence. Category theory was invented as a formal means of establishing commonalities between various mathematical structures. We use category theory to show that transitive inference and class inclusion involve *dual* mathematical structures, called product and coproduct. Other inferential tasks with similar developmental profiles, including matrix completion, cardinality, dimensional changed card sorting, balance-scale (weight-distance integration), and Theory of Mind also involve these structures. By contrast, (co)products are not involved in the behaviours exhibited by younger children on these tasks, or simplified versions that are within their ability. These results point to a fundamental cognitive principle under development during childhood that is the capacity to compute (co)products in the categorical sense.

## Author Summary

Children acquire various reasoning skills during a remarkably similar period of development. Yet, the reasons for these similarities are a mystery. Two examples are Transitive Inference and Class Inclusion, which develop around five years of age. Older children understand that if John is taller than Mary, and Mary is taller than Sue, then John is also taller than Sue. This form of reasoning is called transitive inference. Older children also understand that there are more fruits than apples. This inference is called class inclusion. We explain why these and a variety of other abilities show the same development using a branch of mathematics called category theory. Category theory reveals that they have related underlying structure. So, despite their apparent superficial differences these reasoning abilities have similar profiles of development because they involve related sorts of processes.

## Introduction

Children acquire various reasoning skills over remarkably similar periods of development. Transitive Inference and Class Inclusion are two behaviours among a suite of inferential abilities that have strikingly similar developmental profiles—all are acquired around the age of five years [1]. For example, older children can infer that if *John is taller than Mary*, and *Mary is taller than Sue*, then *John is taller than Sue*. This form of reasoning is called Transitive Inference. Older children also understand that a grocery store will contain more fruit than apples. That is, the number of items belonging to the superclass is greater than the number of items in any one of its subclasses. This form of reasoning is called Class Inclusion. These two types of inference appear to have little in common. Transitive Inference typically involves physical relationships between objects, while Class Inclusion involves abstract relative sizes of object classes. Nonetheless, explicit tests of these and other inferences for a range of age groups revealed that success was attained from about the median age of five years [1].

Since Piaget, decades of research have revealed important clues regarding the development of inference, yet little is known about the reasons underlying these correspondences (see [2] for reviews). A common theme in two recent proposals is the computing of relational information [3],[4]. In regard to *Relational Complexity* theory [3], the correspondence between commonly acquired cognitive behaviours is based on the maximum arity of relations that must be processed (e.g., tasks acquired after age five involve ternary relations, i.e., relations between three items). In regard to *Cognitive Complexity and Control* theory [4], the correspondence is based on the common depth of relation hierarchies. Although a relational approach to cognitive behaviour has a formal basis in relational algebra [5], certain assumptions must be made about the units of analysis. For tasks as diverse in procedure and content as Transitive Inference and Class Inclusion, it is difficult to see how the analysis of one task leads naturally to the other. For Relational Complexity theory, Transitive Inference is considered to involve the integration of *two* binary relations between task elements into an ordered triple, or ternary relation; whereas Class Inclusion is regarded as the integration of *three* binary relations between three sets of elements (one complement and two containments) into a ternary relation [2],[3]. For Cognitive Complexity and Control theory, Transitive Inference involves relations over *items*; whereas Class Inclusion involves relations over *sets of items*.

This theoretical difficulty is symptomatic of the general problem in cognitive science where the basic components of cognition are unknown. In the absence of such detailed knowledge, cognitive modelers have been forced to assume a particular representational format (e.g., symbolic [6], or subsymbolic [7]). This approach, however, does not lend itself to the current problem, because the elements of Transitive Inference and Class Inclusion tasks (i.e., objects and classes of objects) do not share a common basis. Understandably, then, these sorts of behaviours have tended to be studied in detailed isolation, narrowing the scope for identifying general principles.

*Category theory* was born out of a desire to establish formal commonalities between various mathematical structures [8],[9], and has since been applied to the analysis of computational structures in computer science (see [10]–[12]). The seminal insight was a shift from objects as the primary focus of analysis to their transformations. Contrast, for instance, sets defined in terms of (the properties of) the objects they contain—Set Theory—against sets defined in terms of the morphisms that map to or from them—Category Theory [13]. This insight motivates our categorical approach to the analysis of inference, and our way around the current impasse. In cognitive science, several authors have used category theory for a conceptual analysis of space and time [14]–[16], though we know of only one other application that has modeled empirical data [17]. Since our application of category theory to cognitive behaviour is novel, we first introduce the basic category theory constructs needed for our subsequent analysis of Transitive Inference, Class Inclusion, and other paradigms. The analysis begins with a brief introduction of the sort of data our approach is intended to explain, which primarily concerns contrasts between younger and older children relative to age five, and correlations across paradigms. Finally, we extend our categorical approach to more complex levels of inference. Our main point is that, despite the apparent lack of resemblance, all these tasks are formally connected via the categorical (co)product, to be defined below. The significance of this result is that it opens the door to an entirely new (empirical) approach to identifying general principles, particularly in regard to the development of inferential abilities, that are less likely to be revealed by standard modeling methods.

## Methods

In this section, we provide the basic category theory definitions and constructs used in our subsequent analysis of various inferential abilities. Detailed introductions to category theory are found in [12],[18],[19]. Category theory is abstract in the sense that its entities may not refer to particular concrete objects, such as system states, or task stimuli, or even other mathematical objects. This sort of abstractness is a strength of the theory, permitting one to see the formal connection between otherwise disparate fields. Nonetheless, this abstractness may also be a source of bewilderment to those unfamiliar with this approach. Hence, throughout this section, we provide specific examples of category theory concepts for didactic purposes in some cases, and as a prelude to our analysis in others.

### Category

A *category* 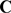 consists of:

- a class 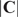 of objects 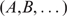;
- a set 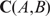 of morphisms (also called arrows, or maps), from 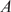 to 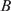, where for each morphism 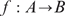 indicates that 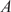 is the *domain*, or *source* and 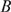 is the *codomain*, or *target* of 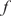 (i.e., 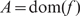 and 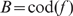);
- a morphism 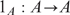 called the *identity* for each object 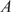; and
- a composition operation, denoted “

”, of morphisms 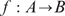 and 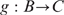, written 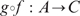, satisfying the laws of:
  - *unity*, where 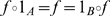, for all 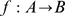; and
  - *associativity*, where 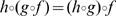, for all 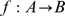, 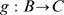 and 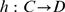.

One immediately recognizable example is the category 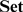, which has sets for objects and functions for morphisms, where the identity morphism 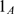 is the identity function and the composition operation is the usual function composition operator “

”. Another, less obvious, example is the category of Euclidean spaces, 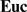, which has 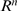 spaces as objects, where 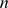 is a natural number; 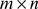 matrices for morphisms 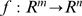, where the identity matrix is the identity morphism; and matrix multiplication is the composition operation. From a cognitive perspective, an object may be a cognitive state, set of states, or some other entity employing symbolic, or numerical representations, and a morphism may be some cognitive process transforming one state to another. At present, we do not prejudge the cognitive nature of objects and morphisms for the reasons already mentioned.

Categories exist for a diverse range of structures, with objects more complex than sets of elements, and structure-preserving morphisms more complex than associations. For example, the following morphism
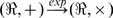
(1)maps from object 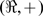 to object 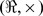, where each object consists of the set of real numbers with additional internal structure (i.e., a rule—respectively, addition and multiplication—for combining two numbers into another number). This morphism maps real numbers 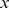 to 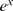, where “+” in the domain corresponds to “
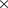
“ in the codomain. Structure is preserved by 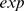, because the transformation of the result of applying the rule to the numbers is the same as the result of applying the corresponding rule to the transformed numbers. In this case, 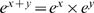, for all 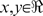. This morphism and its (co)domain objects are members of the category of semigroups, which has semigroups for objects and semigroup homomorphisms for arrows. A semigroup is just a set 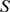 with an associative binary operation 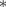, and a semigroup homomorphism, 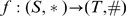, preserves an object's internal structure as illustrated: that is, 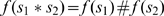. Hence, 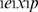 is a semigroup homomorphism. But, not every function is a morphism in this category. For example, 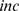 (i.e., increment by 1)
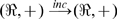
(2)is not a semigroup homomorphism, because 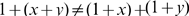. These examples illustrate that although category theory is abstract, it is not arbitrary. So, statements derived from the theory are, in principle, testable and falsifiable.

### Dual

We need to introduce the notion of the *dual* 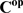 of a category 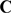. 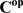 is essentially 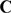 with all arrows reversed. That is, the set of objects in 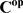 is the same as in 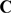; there is a one-to-one correspondence between arrows in 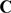 and 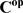 such that the arrow 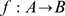 in 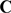 corresponds to the arrow 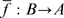 in 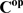; and the composition 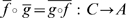 is defined in 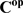 exactly when 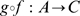 is defined in 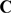. A definition or a proposition and proof about 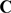 gives rise to its dual in 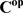 by taking the original definition/proposition/proof and reversing all the arrows. Any valid argument about the arrows in 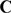 is valid for the dual argument in 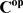, so proving or defining something in 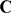 gives you something for free in 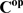. Obviously, reversing an arrow twice will return the original arrow, so 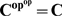.

Some examples of duals involve certain types of morphisms, called epimorphisms, monomorphisms and isomorphisms. A morphism 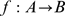 is an *epimorphism*, if for any pair of morphisms 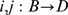, 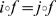 implies 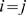. That is, 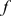 is an epimorphism if whenever the following diagram *commutes*, 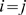,
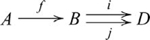
(3)(Commutative diagrams afford proof by *arrow chasing*. A diagram is said to be commutative if the compositions of the morphisms on any two paths through the diagram, from a common start object to a common finish object, are equal, except when both paths are of length 1. In Diagram 3, the start object is 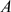, the finish object is 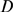, and the two paths are 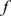, 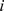 and 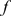, 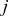.) For 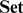 and 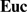, a morphism 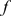 is an epimorphism if and only if it is onto (i.e., informally, there are no elements in the codomain that are unreachable from elements in the domain via 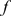). A morphism 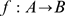 is a *monomorphism*, if for any pair of morphisms 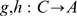, 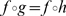 implies 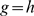,
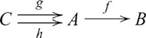
(4)For 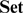 and 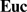, a morphism 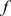 is a monomorphism if and only if it is one-to-one. By reversing arrows, we see that the definition of an epimorphism in a category 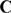 is the definition of a monomorphism in the category 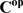 (i.e., the definitions are dual). A morphism 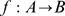 is an *isomorphism* if there exists a morphism 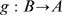, such that 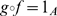 and 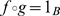. An isomorphism in 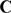 is also an isomorphism in 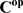. A more formal treatment of duality can be found in Text S1.

### Products

Cognitive behavior generally involves some means of integrating information. A general notion of integration is the categorical product. In any category 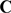, a *product* of two objects 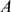 and 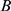 is an object 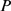 together with two morphisms 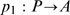 and 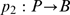, such that for any pair of morphisms 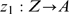 and 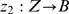, there is a unique morphism 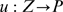, such that the following diagram commutes
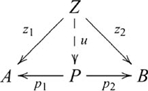
(5)where a broken arrow indicates that there exists exactly one morphism making the diagram commute. The morphisms used in the definition of a product (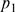 and 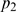 above) are sometimes called projection morphisms. A product object 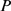 is *unique up to a unique isomorphism*. That is, for any other product object 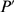 with morphisms 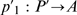 and 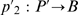 there is one and only one isomorphism between 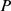 and 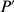 that makes a diagram like the one above commute. This means that 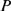 is not unique, only unique with respect to another product object via isomorphism (a point to which we will return shortly), which is why the definition refers to *a* product, not *the* product. An essential characteristic of a product object is that the constituents 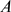 and 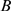 are retrievable via the projection morphisms. 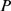 is also written 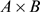, and since 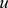 is uniquely determined by 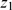 and 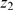, 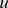 is often written as 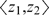, and the diagram used in defining a product then becomes
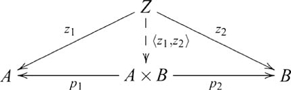
(6)


In 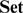, 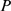 is, up to isomorphism, the Cartesian product 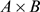, 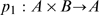, 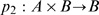, where 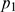 and 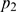 are the projection maps to 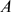 and 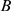, i.e., 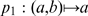, and 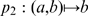, and 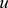 is the product function 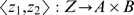, sending 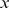 to tuple 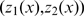, so that 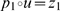 and 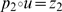. (The 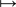 arrow, often read as “maps to”, indicates the action of a function on a domain element. Thus 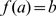 is equivalent to 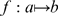.) For example, suppose 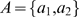 and 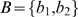, then 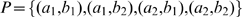, and 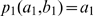, 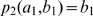, and so on. Suppose 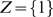, 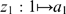, and 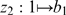, then the only morphism 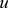 making this example commute is 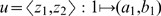.

One can think of tasks involving stimuli that vary along two task-relevant dimensions as examples involving categorical products. For example, classification tasks where the rule is based on, say, stimulus colour and size involves a product, with the set of task stimuli as the product object and the determination of colour and size features as the projection morphisms. Conservation tasks, for example, predicting whether the amount of liquid in one container is the same as another where the containers vary in, say, height and width also involve products. In this case, the product object is a set of volumes and the projection maps recover the associated heights and widths. We will see further examples of tasks involving products in the next section.

For our purposes, the categorical product 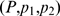 is a statement about a cognitive (sub)system, whereas the triple 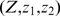 is a constraint on what constitutes a valid product rather than a specific claim about cognition. Notice that 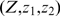 is not necessarily a product in its own right, as the example above illustrated, since the one element set 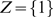 is not isomorphic to the Cartesian product containing four elements. Notice, further, that although 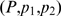 does pertain to the cognitive system it is not a commitment to a particular representation and process. To illustrate, the product object in the previous example could just as easily be defined as the set 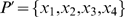, without explicitly identifying the components 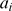 and 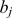, so long as the projections 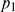 and 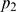 recover those components appropriately. From the categorical perspective, these two isomorphic alternatives are the “same”, relieving us of any prior commitment to, say, classical [20] or functional [21] compositionality, which has been a contentious issue when framing theories of cognition [22].

### Coproducts (sums)

A related notion of information integration is the categorical coproduct. In any category 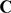, a *coproduct* (or, *sum*) of two objects 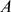 and 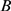 is an object 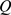 together with two morphisms 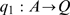 and 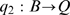, such that for any pair of morphisms 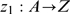 and 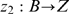, there is a unique morphism 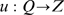, such that the following diagram commutes
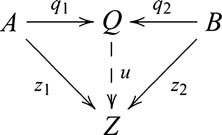
(7) The morphisms used in the definition of a coproduct (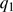 and 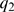 above) are sometimes called injection morphisms. A coproduct object 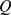 is also unique up to a unique isomorphism. 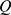 is also written 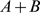, and since 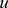 is uniquely determined by 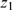 and 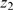, 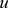 is often written as 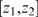, and so the coproduct diagram becomes
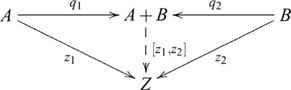
(8)


In 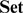, 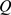 is the *disjoint union* 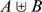, 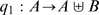, and 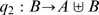. Suppose, for example, 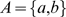 and 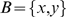, then 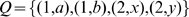. Basically, every element in 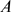 and 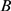 is augmented with a label that identifies the set from which it came. Unlike set union, which removes duplicates, all information is maintained.

If we reverse all the arrows in the definition of a coproduct we get a product. A product in a category 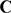 is a coproduct in 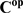. Coproducts are dual to products. The duality between product and coproduct is shown formally in Text S1.

One way to think about coproducts in terms of cognitive tasks is to regard the label as the context or condition under which a stimulus is associated with a particular action. Experimental paradigms designed to assess cognitive flexibility, such as the Wisconsin Card Sorting Task, are examples. For instance, in one context, say, a reward schedule based on colour, a red triangle may require one type of response, but for a reward schedule based on shape, the red triangle requires a different type of response. In this case, the coproduct object is the disjoint union of the stimulus set with itself with colour and shape as labels, and the response is determined by a map from the coproduct object to a set of actions.

### Pullbacks and pushouts

More generally, information integration is often subject to satisfying some constraint. Hence, product and coproduct are instances of more general constructs known as pullbacks and pushouts, respectively. A *pullback* of morphisms 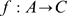 and 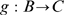 is an object 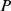 and a pair of morphisms 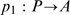 and 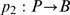 satisfying 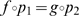, such that for any pair of morphisms 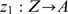 and 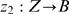 such that 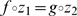, there is a unique morphism 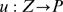, such that the following diagram commutes:
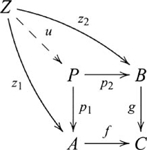
(9)In such a pullback, 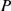 may also be denoted by 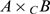. The constraint is contained in the requirement that the square in Diagram 9 should commute.

Intersection is an example of pullback in 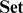, where 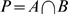, 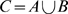, and 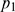, 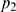, 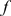 and 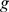 are inclusions. More generally, a pullback is a constrained product, restricted to satisfy the constraints imposed by 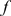 and 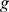, so that 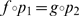, with 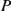 as the set of solutions.

Pushout is dual to pullback. A *pushout* of morphisms 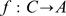 and 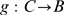 is an object 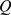 and a pair of morphisms 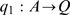 and 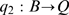 satisfying 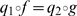, such that for any pair of morphisms 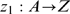 and 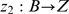 such that 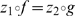, there is a unique morphism 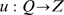, such that the following diagram commutes:
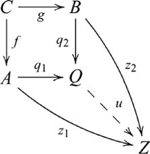
(10)In such a pushout, 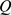 may also be denoted by 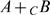.

Given the duality, union is an example of pushout in 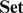, where 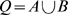, 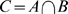, and all morphisms are inclusions. In this case, the pushout is also a pullback. A more general pushout in 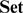 involves a form of disjoint union such that elements 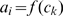 and 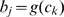 are *identified* (i.e., “glued” together) in the pushout object [9],[23]. For example, suppose 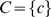, 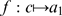 and 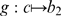, and 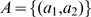 and 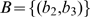, then 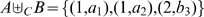 or, equivalently, 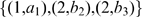. Here 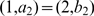, because they have been identified as described above. (These elements are actually equivalence classes whose members are identified by *coequalizers* [24], but we use this form for convenience.) In general, 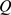 is the integration of components providing no more and no less information than necessary to satisfy the requirement that 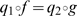.

For our purposes, the commutative squares in the pullback and pushout diagrams pertain to statements about cognitive (sub)systems, and 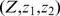 constrains what constitutes a valid pullback/pushout construction. Aside from definitions, then, we no longer refer to 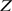 and associated morphisms 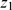, 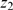, and 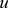, so they are omitted from subsequent diagrams.

### Initial and terminal objects and their (co)products

An *initial* object in a category 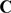 is an object 0, such that for every object 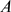 there is exactly one morphism 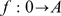 in 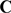. A *terminal* object is an object 1, such that for every object 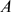 there exists a unique morphism 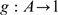 in 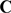. In 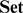, the only initial object is the empty set 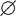, and any one-element set, e.g., 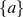, is a terminal object. (In 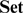, the initial object, 0, has 0 members, while the terminal object, 1, has 1 member. In some other categories, e.g. 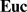, the initial object is also the terminal object, and is then called a null object. Some categories, such as a discrete category with no non-identity arrows, lack an initial object, or a terminal object, or both.) Multiple initial objects in a category are not distinguished because they are isomorphic, and the same also applies to terminal objects [18],[24]. In a category with initial and terminal objects, products and coproducts, a product of an object 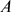 with a terminal object is isomorphic to 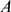, 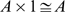; and a coproduct of 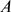 with an initial object is isomorphic to 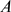, 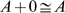 [18],[24]. For example, the Cartesian product 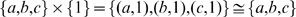; and the disjoint union 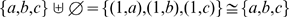. The following diagram
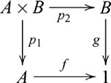
(11)involving a terminal object (1) is always a pullback, and when 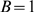, 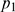 is an isomorphism. The following diagram
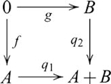
(12)involving an initial object (0) is always a pushout, and when 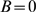, 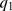 is an isomorphism. For subsequent pullback/pushout diagrams, we omit references to specific morphisms with an initial object as its domain, or a terminal object as its codomain, since their existence is guaranteed by definition. Usually, the initial or terminal object is also omitted in these cases, but we choose to show it for conformity with the other diagrams.

These “special” cases are important for determining whether a system that apparently involves a (co)product is in fact isomorphic to one that does not. We will see an example of this situation in the next section. In these situations, we say that task difficulty is related to the simpler, non-(co)product form.

Notice that we could have explained all this just in terms of the particular product, coproduct, pullback, pushout, initial and terminal object that prevail in 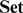. Presenting in the more general case of products, etc., in an arbitrary category, makes it clearer that these are not constructions specific to 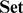, but instances of a wider phenomenon.

## Results

In this section, we apply category theory concepts to the analysis of results from several studies that have provided empirical evidence of within group similarities and between group differences in behavioural performance across multiple tasks. The objective is to identify a formal basis for an equivalence class of tasks that accounts for these similarities and differences. Our seed paradigms are Transitive Inference and Class Inclusion, which were tested on age groups ranging from three to eight years [1]. The main finding was that significant above chance performances on Transitive Inference and Class Inclusion were observed around five years. By contrast, younger children (three- and four-year-olds) showed significant above chance performance only on simpler versions of these two tasks. The thrust of our analysis is to show that these tasks are formally connected by the dual relationship between product (Transitive Inference) and coproduct (Class Inclusion). By contrast, the simpler versions of these tasks do not involve a (co)product, or involve a (co)product in the trivial sense (e.g., 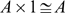). Then, in the remainder of this section, we extend this analysis to other paradigms, including: Matrix Completion, Cardinality, Card Sorting, Balance-scale, and Theory of Mind. In each case, the more difficult version of the task, where significant above chance performance was observed in the five-year-olds and older children, involves a product or coproduct. By contrast, performance in the younger age groups does not, or involves a (co)product with an initial or terminal object, which reduces via isomorphism to a single map. Thus, the core characteristic that distinguishes performance by younger (three-, or four-year-olds) versus older (five-year-olds and above) children is computing the categorical (co)product and its encompassing pullback (pushout).

The data of primary concern here are the correlations in achievement across paradigms and the significant differences between age groups within paradigms. Age five is regarded as a “nominal” timepoint in that some children exhibit success at a younger or older age. For example, 11% of the three- and four-year-olds, and 71% of six-year-olds succeeded on Transitive Inference, and respectively 15% and 67% succeeded on Class Inclusion [1] (Table 16). Correlations are directly testable using within-participant across-paradigm studies. For this reason, our primary data sources come from three studies showing significant correlations between Transitive Inference, Class Inclusion, and Cardinality [1]; Transitive Inference, Class Inclusion, Cardinality and Theory of Mind [25]; and Transitive Inference, Class Inclusion, and Balance-scale[26]. For example, the correlations in performance between Transitive Inference and Class Inclusion (0.54), Transitive Inference and Cardinality (0.54), and Class Inclusion and Cardinality (0.47) were all significant (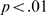) [1] (Table 6). However, to illustrate the diverse applicability of our category theory approach, we also include two other contrast studies revealing significant differences between the same age groups (i.e., younger versus older than age five) albeit with different individuals for Matrix Completion (see [2]), and Dimensional Change Card Sorting [27].

### Transitive Inference

A transitive inference has the general form that given 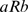 and 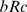, then one can infer 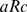, where 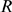 is some binary relation that has the transitivity property. A Transitive Inference task, as typically administered to children, involves presenting participants with a series of premise pairs followed by a series of test pairs to assess inferential capability. The premise series usually consists of four pairs, AB, BC, CD, and DE, and testing is done on non-adjacent pair, BD (not in the premise series). AC and DE are not considered as evidence of transitive inference, because a consistent response is obtainable by noting that A or E was paired with only one other stimulus.

Transitivity is a property of relations, so a transitive inference is just a particular operation in relational algebra. In relational algebra, an *equijoin* of two relations is the set of tuples that have the same values on the specified attributes. For example, suppose 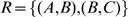 and 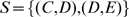, then the equijoin along the second and first attributes of 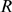 and 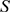 (respectively) is 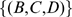. (Only tuples with the same values at the specified attributes are joined, and the redundant attribute removed. For further details of relational operators, see for example [28].) A transitive inference, then, involves a product of premise relations, indicated in the following example diagram
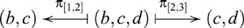
(13)where 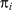 is the *project* operator in relational algebra, returning the values of each relation instance at the attributes listed by 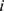. The transitive inference 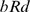 involves the constraint that premises involving 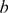 and 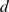 share a common element 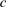 in the second and first positions, respectively (i.e., 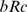 and 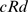). This constraint is captured by the following diagram (which relates to a pullback)
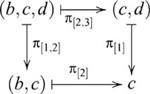
(14)where the joins of other premise pairs (e.g., AB and BC based on the common element B) are omitted for clarity.

To contrast younger versus older children's performance, children were presented with difficult and simple versions of this paradigm [1]. In the difficult version of the task, children were presented with two non-adjacent blocks BD and asked which block would be higher in a tower constructed from the premise pairs (mini-towers). From Diagram 14 we see that this inference involves a map from the product object. That is, given 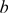 as one block, then the other block will be higher if it corresponds to 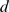. In the simpler version of the task, children were given one of the mini-towers and a sequence of adjacent blocks and asked to build the complete tower (e.g., BC, followed by D, A, E). Each step only requires a map from one of the premise objects to determine where the next block should be placed. Thus, it does not require computing the product. Significantly, while younger and older children were successful on the simpler version of the task, the older children but not the younger ones were generally successful on the difficult version [1].

### Class Inclusion

In a Class Inclusion task, participants are given examples of a superclass, and two complementary subclasses and asked about their relative sizes. For example, given the superclass, *fruit*, and subclasses *apples* and *non-apples*, participants are asked, *Are there more apples than fruit?* We show that class inclusion involves a coproduct. Coproduct is the dual of product, hence there is a duality between Class Inclusion and Transitive Inference.

Class inclusion is a property of sets, so a class inclusion inference involves a particular set operation—disjoint union. As we have seen, the disjoint union of two objects in the category of sets is the coproduct. Suppose, for example, the set of apple referents, or indices 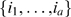 and non-apple indices 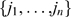. The coproduct is
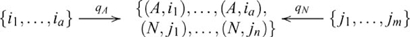
(15)where 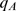 and 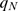 are the apple and non-apple injection maps, respectively. The inference is obtained by observing the cardinality of each set. Typically, a Class Inclusion task involves complementary subsets, so their intersection is empty. This arrangement is captured in the following pushout diagram
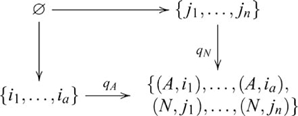
(16)In a variation of Class Inclusion where 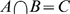, elements common to subclasses 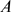 and 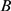 would be identified by 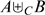, so they would not be counted twice in the superclass, i.e., 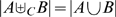.

The same groups of children who were tested on Transitive Inference were also tested on Class Inclusion [1]. Three questions were posed to children who performed a version of Class Inclusion consisting of blue triangles and circles, so that the two subclasses were triangles and circles and the superclass was blue shapes. They were: (1) *Are there more triangles than circles?* (2) *Are there more blue things or more triangles?* (3) *Are there more circles or more blue things?* The older children were successful on all three questions, whereas the younger children were generally successful on the first question only [1]. Questions 2 and 3 involve maps from one of the component objects and the coproduct object to their cardinalities. By contrast, Question 1 involves maps from the component objects only, so the coproduct object is not involved.

There is a subtle difference between the diagrams for Transitive Inference and Class Inclusion. Transitive Inference involves a *constrained* product, while Class Inclusion involves an *unconstrained* coproduct. The bottom-right object in Diagram 14 is not a terminal object (other constraining elements were omitted), whereas the top-left object in Diagram 16 is the initial object. This difference has implications for pullback/pushout diagrams containing (co)products of more than two objects, which we address in the next section when we consider a more complex version of Class Inclusion. The other paradigms considered in the remainder of this section involve only unconstrained (co)products.

### Other paradigms

Transitive Inference and Class Inclusion are both difficult for children below about the age of five years. Our analysis indicates that underlying this common difficulty is a lack of capacity to compute categorical (co)products. In the remainder of this section, we analyze other tasks used to compare performance within and contrast performance between groups of younger and older children.

#### Matrix Completion

In a modified version of Matrix Completion [2], children are presented with a grid of figures that vary along rows and columns in either one or two feature dimensions (e.g., colour and shape). The task is to infer a missing figure that matches features with the other figures in the corresponding row and column. For example, if the first, second and third rows contain circles, triangles and squares; and the first, second and third columns contain red, green and blue figures (respectively), then the missing figure in the second row and column is a green triangle. This inference is obtained from a product of the object containing the shapes and the object containing colours. The pullback is indicated by the following diagram
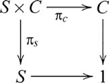
(17)where 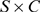 is the Cartesian product of the set of shapes 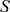 and colours 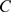; 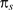 and 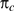 are the shape and colour projections; and 1 is terminal (i.e., there are no constraints on the product). The grid identifying shape locations is also construed as a product, 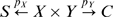, where 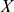 and 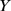 are the row and column positions in the grid and 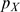 and 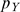 are the respective index to shape and colour maps. We regard this product as input to the cognitive system—thus 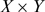 takes the roll of 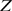 in the definition of product. The defining property of a product guarantees the existence of 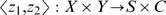, and 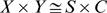 (see Products), so the correct coloured shape can be inferred from the missing figure location. Older children have demonstrated successful performance on this task. However, younger children were generally shown to be capable of only simpler forms of this task where one of the feature dimensions was constant (e.g., all red shapes, or all triangles, see [2]). In the simpler forms of this task, one of the component objects contains only one element. For example, if all shapes were coloured red, then the colour object is the set 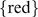. As indicated in the previous section, a single element set is a terminal object in the category 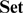. The product object as indicated in the following diagram
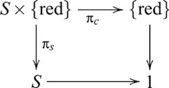
(18)is isomorphic to the shape object, 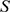, and therefore not required for the inference. The correct figure is inferred by a direct map to the shape object (i.e., 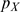). Since all terminal objects are isomorphic, the same result is obtained regardless of the particular feature held constant, as we would expect.

#### Cardinality

Participants are presented with rows of items (e.g., four ducks, five frogs, or seven balls), and are asked three types of questions: (1) *How many x are there?* where 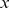 identifies the type of item (e.g., ducks); (2) *Can you show me y by drawing a circle around y?* where 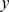 is the number of items counted; and (3) *If you counted from the other end, how many would there be?* The third question directly tests the understanding that counting does not depend on order. Thus, a counting strategy that simply increments a counter in a particular order would fail when the order was reversed. A counting strategy that does not rely on item order must keep track of two types of items: those that have been counted; and those that have not. These two types are complementary subsets of the total set of items to be counted. Hence, Cardinality involves the same pushout structure as Class Inclusion, as indicated by the following diagram
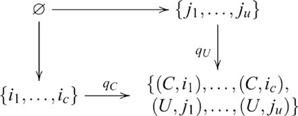
(19)where 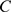 and 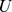 refer to the counted and uncounted subsets, respectively. In general, four-year-olds were successful on the first two questions, but only the older groups (five years and above) were successful on the third [1]. Questions 1 and 2 do not require anything more than in-order counting of a set of elements already identified by a single type or location. Hence, counting in these two situations does not involve constructing a coproduct of subtypes. Although this task arguably has an added component where elements are transferred from the uncounted to the counted set, it is not part of the coproduct process.

#### Dimensional change card sorting

In this task, participants are presented cards identifiable by coloured shapes on the visible side. Two target cards are placed on a table. Children play two sorting games by placing additional (sort) cards under one of the target cards based on the same colour (colour game), or same shape (shape game). Suppose, for example, the target cards were labeled *red triangle* (▾) and *green circle* (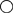). In the context of playing the colour game, a child should place a *red circle* (•) card under the *red triangle* target card, and a *green triangle* (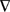) card under the *green circle* target card. In the context of the shape game, a *red circle* card should be placed under the *green circle* target, and a *green triangle* card under the *red triangle* target. In general, only the five- and six-year-olds performed this task above chance level, but not the three- and four-year-olds [27]. By contrast, three- and four-year-olds were only successful on a simpler form of the task where one of the two feature dimensions was constant. For example, in the shape game, the additional cards were either red triangle or red circle cards. The more difficult version involves a product between dimension (colour, shape) and the two sort cards (•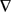) as indicated by the following diagram
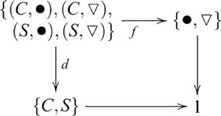
(20)where 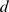 and 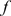 are the dimension and figure projections (respectively). The inference is obtained by a map from the product object to the object containing the two target cards (not shown). In the simpler version of this task, the sort cards differ on only one dimension, so the games involve different sets of sort cards. For the colour game, the sort cards are • and 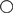; and for the shape game, • and ▾. In this case, the dimension and sort card objects do not form a *correct* product—because the sort card object now has three elements, the product contains pairs (*C*,▾) and 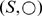 that were not part of the task. Rather, in each game, the target is inferred from just one feature dimension, either colour or shape but not both. Hence, the simpler version does not involve a product.

#### Balance-scale: weight-distance integration

In a modified form of the balance-scale task, called weight-distance integration, participants were shown a one-arm balance and asked to predict the degree of tilt given a weight placed at a distance from the pivot [26]. The arm was horizontal when no weight was added, and pointed to one of nine pictured animals when tilted. Thus, a tilt angle prediction was indicated by the expected animal pointed to after releasing the arm. A spring provided the appropriate balance mechanism. There were three levels of weight and three levels of distance. Five-, six- and seven-year-olds predicted tilt by integrating both distance and weight information, either additively or multiplicatively. Four-year-olds predicted tilt without integration (i.e., by weight, or distance only). Three-year-olds used neither strategy and were generally non-systematic in their responses [26]. Since product and coproduct are generalizations of multiplication and addition, then the integrative strategies are naturally captured by pullbacks and pushouts, respectively. The pullback is indicated by the diagram
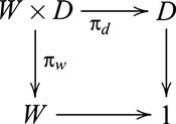
(21)where 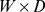 is the Cartesian product of the set of weights 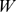 and distances 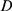; 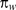 and 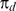 are the weight and distance projections; and 1 is terminal (i.e., there are no constraints on the product). Tilt prediction is a map 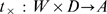 from the product object to the set of animals indicating the degree of tilt (not shown). The additive strategy is captured by the following pushout diagram
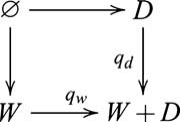
(22)where 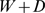 is the disjoint union of weight and distance sets; and 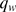 and 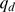 are the corresponding injections. Tilt prediction is a map 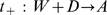 (not shown). The simpler strategy used by the four-year-olds just involves a map from either the weight or distance object to the predicted tilt position.

#### Theory of mind

For this paradigm two sorts of tasks were employed: appearance-reality; and false-belief [25]. As an example of an appearance-reality task, children are shown milk poured into a glass wrapped in a red filter. The colour of the milk is visible before and after being poured into the glass. After filling the glass they are asked an appearance question, *When you look at this milk right now, does it look red or does it look white?*; and a reality question *What color is the milk really and truly? Is it white or is it red?* As an example of a false-belief task, children are shown drawings of a boy and a puppy and told the story, *Sam wants to find his puppy. Sam's puppy is really in the kitchen. Sam thinks his puppy is in the bathroom*. They are then asked a belief question, *Where will Sam look first for his puppy, the bathroom or the kitchen?*; and a reality question, *Where is the puppy really, the bathroom or the kitchen?* Four variations of appearance-reality and four variations of false-belief tasks were tested on three age groups: three-, four- and five-year-olds (16 participants in each group). Only one of the three-year-olds, seven of the four-year-olds and twelve of the five-year-olds passed the combined tasks, where pooled responses over the eight tasks were significantly above chance [25]. Both appearance-reality and false-belief involve a pushout. The following diagram indicates the pushout for the milk task example
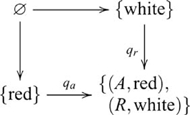
(23)where 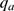 and 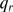 are the injection maps, identifying colour in the context of appearance and reality (respectively). The puppy task example is similarly indicated by the following pushout diagram
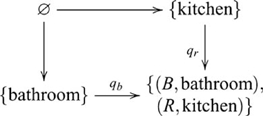
(24)where 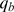 and 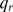 are the injection maps, identifying location in the context of belief and reality (respectively). In both cases, the inference is obtained by a map from the coproduct object to colour or location. By contrast, without the coproduct object one cannot determine the correct context for colour or location. Hence, the expected number of correct responses is the same as chance, corresponding to the performance of the younger children.

### Summary

The distinguishing characteristic at the heart of the behavioural difference between younger (less than five years old) versus older (more than five years old) children is the categorical (co)product. In the case of Transitive Inference, Matrix Completion, and Card Sorting, this difference was realised by task design (e.g., one versus two relevant feature dimensions). In the case of Class Inclusion and Cardinality, this difference was realized by questions probing, for example, one versus two feature dimensions. And, in the case of Balance-scale and Theory of Mind, this difference was realized by alternative task strategies as inferred from the types of response errors. In each paradigm, the more difficult situation observed in the older children required access to a (co)product. By contrast, the less difficult situation observed in younger and older children involved directly accessing the component objects without computing or accessing a (co)product. These correspondences have been confirmed directly with the same participants performing multiple paradigms that included: Transitive Inference, Class Inclusion, and Cardinality [1]; Transitive Inference, Class Inclusion, Cardinality and Theory of Mind [25]; and Transitive Inference, Class Inclusion, and Balance-scale [26].

### Beyond early development: Finite (co)products

So far, our analysis has been confined to early development around the age of five, where the capacity to compute (co)products was identified as crucial. The more interesting statistic for our purposes is the correlation across paradigms, rather than a specific age of attainment. That is, for example, whether or not a four(six)-year-old who succeeds (fails) at Transitive Inference also succeeds (fails) at Class Inclusion. However, the simpler versions of these tasks often form baselines that are within the capacity of all children. In these situations, *floor* effects may attenuate the ability to detect significant correlations. A methodological solution is to contrast tasks at “higher levels” of complexity at which neither level constituents a baseline (i.e., within the capacity of all participants). Hence, in this section, we extend our analysis to more complex tasks.

A number of studies point to higher complexity levels, at least in adult cognition. For example, adults were tested on their ability to identify the number of interactions underlying fictitious data sets reported as bar graphs [29]. A two-way interaction, for instance, was identifiable by observing that the change in bar height between conditions 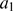 and 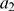 under condition 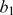 differed under condition 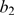. The maximum number of interactions that adults could effectively recognize was about four [29]. Adults have also been tested on Raven's Progressive Matrices, which is closely related to Matrix Completion, where the number of feature dimensions was increased to three. Functional magnetic resonance imaging revealed significant differences in activity for regions in the prefrontal cortex when figures varied along three versus two feature dimensions [30]. These sorts of tasks have been characterized in terms of the arity of relations processed (e.g., binary, ternary, quaternary) [3], or the number of related task “variables” [31]. Our main purpose in this section is to show how our category theory approach incorporates higher levels of complexity. Unlike the studies examined in the previous section, there have not been multi-paradigm within-participant comparison/between-participant contrast studies for these more complex tasks. So, we proceed by extending the analysis to more complex versions of the seven paradigms considered above.

In category theory, the (co)product extends naturally to any finite number of objects. Moreover, the degenerate case where the number of objects is one corresponds to the *no (co)product* cases in the previous section. First, we provide the basic definitions before showing how the existing paradigms are extendable to more complex cases.

#### Finite (co)products

In any category 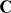, a *finite product* of 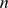 objects 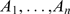 is an object 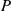 together with 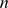 morphisms 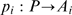, such that for any 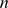-tuple of morphisms 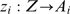, there is a unique morphism 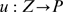, such that the following diagram commutes
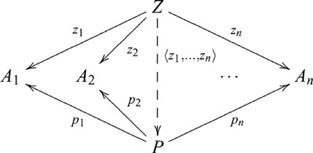
(25)where 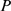 is denoted by 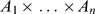, and 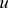 by 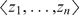.

The finite coproduct is defined similarly. In any category 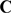, a *finite coproduct* of 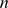 objects 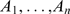 is an object 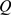 together with 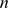 morphisms 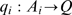, such that for any 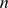-tuple of morphisms 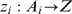, there is a unique morphism 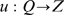, such that the following diagram commutes
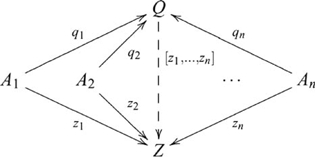
(26)where 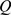 is denoted by 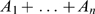, and 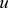 by 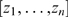. Naturally, finite product and finite coproduct are dual. That is, a coproduct of 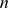 objects in 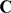 is a product of 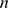 objects in 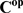.

There are four ways of constructing a product of three objects, and the product objects, though not equal, are isomorphic, i.e., 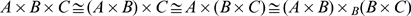. The construction 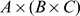 is indicated in the following diagram
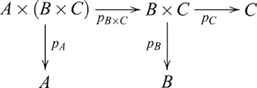
(27)The diagram for the construction 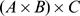 is similar except that the product objects are arranged vertically. However, the construction of 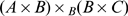 also involves a pullback, as indicated in the following diagram
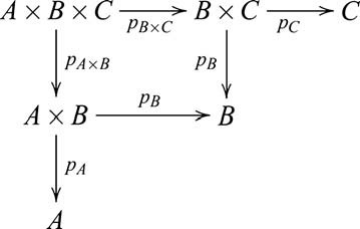
(28)where the ternary product object is denoted 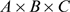, since the four ternary product objects are isomorphic.

There are also four ways of constructing a coproduct of three objects, and the coproduct objects are also isomorphic, i.e., 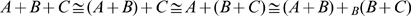. These constructions are dual to the respective products. The construction of 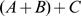 is indicated by the diagram
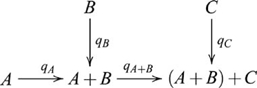
(29)and 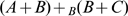, which contains a pushout, is indicated by the diagram
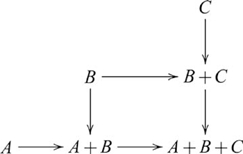
(30)where the ternary coproduct is denoted 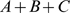.

#### Extensions to paradigms

All seven paradigms analyzed in the previous section can be extended in terms of (co)products of more than two objects. Only ternary (co)products are considered here, but extensions to more objects are also possible. We focus on Transitive Inference and Class Inclusion, and sketch extensions to the other paradigms. Transitive Inference can be extended to include an additional premise EF, and an additional nonadjacent test pair BE that requires two equijoins, for example, BC and CD to infer BD, and BD and DE to infer BE. In category theory terms, this inference involves three pullbacks, indicated by the diagram
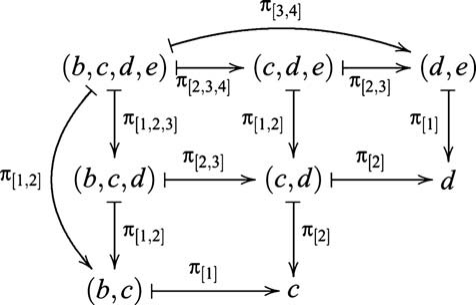
(31)omitting some composition morphisms for clarity. By the *pullback lemma* [24], if two adjacent commutative squares are pullbacks, then the composed *commutative rectangle* is a pullback. This diagram contains two commutative rectangles, indicated in part by the composition arrows 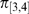 and 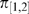.

Class Inclusion can be extended dually by supposing an additional subclass (e.g., squares). For example, participants are presented with small blue triangles (T), small red circles (C) and large red squares (S). They are asked: (1) *Are there more triangles and circles than red shapes?* (2) *Are there more triangles and circles than shapes?* (3) *Are there more circles and squares than shapes?* Question 1 involves binary coproducts, whereas Questions 2 and 3 involve ternary coproducts. For comparison with Question 1 of the original Class Inclusion task, the question *Are there more triangles or circles?* involves neither binary nor ternary coproduct, or what might be called a unary coproduct. The ternary coproduct is indicated in the diagram
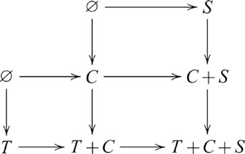
(32)


Notice that although this diagram involves a ternary coproduct, which is dual to a ternary product, the diagram itself is not dual to Diagram 31 for extended Transitive Inference. The reason is that the two initial objects in the extended Class Inclusion diagram are the same (and so too are the two morphisms with 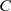 as their codomain). Since the diagrams do not have the same number of objects (i.e., eight versus seven), they cannot be isomorphic. This difference, which we touched on earlier, arises because the coproduct is unconstrained.

An alternative version of extended Class Inclusion that uses constrained coproducts involves subclasses containing common elements. For example, suppose that instead we have a collection of small and large rectangular bars of various colours and orientations. Within this collection, three subclasses are relevant: small (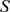), red (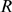) and vertical (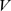) bars, which include small red (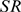) and red vertical (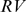) bars. The following corresponding questions are asked: (1) *Are there more small bars and red bars than red bars and vertical bars?* (2) *Are there more small bars and red bars than bars?* (3) *Are there more red bars and vertical bars than bars?* The constrained coproducts are indicated in the following diagram.
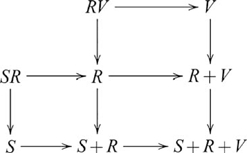
(33)where 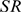 and 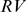 are not the same object. Hence, this diagram is dual to the extended Transitive Inference diagram.

Matrix Completion, Dimensional Change Card Sorting, and Balance-scale involve similar extensions to ternary products, though the latter two are redesigned to accommodate all three levels of products (i.e., unary, binary, and ternary) within the one paradigm. For Matrix Completion, the figures vary along a third feature dimension, such as size. In this case, the task involves a ternary product of colour, shape and size, i.e., 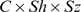. Dimensional Change Card Sorting can be modified to include a third *switch* rule indicating whether the cards are to be sorted using the same (no switch) or opposite (switch) colour or shape feature. In this case, the task involves a ternary product of rule, dimension and shape, i.e., 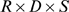. The Balance-scale task can be modified so that tilt angle also depends on spring strength. This case involves a product of the spring (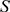), weight and distance, i.e., 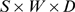.

Theory of Mind and Cardinality involve more substantial changes, so we address these two tasks separately. Theory of Mind can be extended by including an additional transformation condition that involves mixing powered chocolate, which changes the colour of milk to brown. In this case, there are two binary coproducts for separately combining the reality and filtered glass contexts, and reality and mixing contexts, and one ternary coproduct for combining all three contexts, as indicated by the following diagram
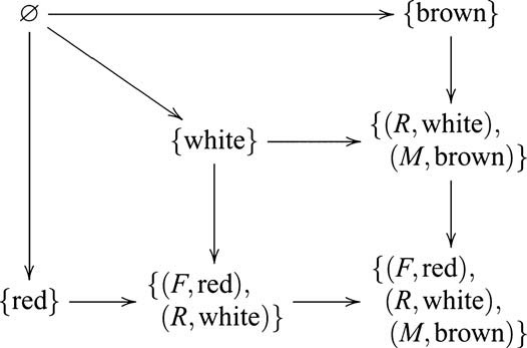
(34)where 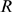 is the reality context, 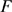 is the filter context, and 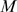 is the mixing context, and prefixing for the ternary coproduct elements is omitted for clarity. This diagram, like Diagram 32, involves unconstrained coproducts. It is isomorphic to the unconstrained version of extended Class Inclusion (Diagram 32), but not the constrained version (Diagram 33), or extended Transitive Inference (Diagram 31). Unlike Class Inclusion, though, there does not appear to be a constrained coproduct version of Theory of Mind in this form, since there is only one object of interest (e.g., milk) in the task.

Cardinality in its current form, though, does not appear to have an extension to ternary coproducts. A possible alternative form, similar to extended Class Inclusion, requires participants to count various combinations subclasses/superclasses (e.g., triangles, circle and squares). The diagram for this case involves a ternary coproduct like the one for unconstrained or constrained Class Inclusion (see Diagram 32 and 33), where the objects are sets of indices and the coproduct is disjoint union (see Diagram 16).

#### Segmenting and chunking

For some tasks, there may exist alternative task strategies for achieving the same goals without exceeding capacity limits. In the context of Relational Complexity Theory, two general strategies were identified as segmenting and chunking [3]. *Segmenting* refers to serializing a process by computing intermediate results. In fact, an example of segmenting appeared in the previous section as the simplified version of Transitive Inference, where complete premise towers were constructed from a sequence of mini-towers, circumventing the binary product. The key to this strategy was supplied by the experimenter in the form of adjacent blocks. *Chunking* refers to recoding information to temporarily reduce (relational) details. Labeling is a common strategy. For example, the relationships between two hydrogen and one oxygen atom are chunked as water. A key to this strategy is learning. Here, we show the category theory equivalent for the extended versions of Transitive Inference and Class Inclusion.

From the definitions, we saw four ways of constructing ternary (co)product objects. Although these objects are isomorphic, Diagram 27 and 28 for constructing 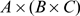 and 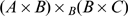 are not, since the first refers to five objects, but the second refers to six. This difference suggests an alternative diagram for computing Transitive Inference BE from premises BC, CD, and DE. Indeed, in the following diagram
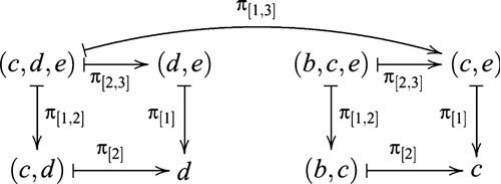
(35)the inference BE is derived by mapping out elements 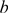 and 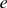 from the product object 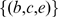 in the right pullback with the morphism 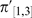 (not shown). Notice that the two pullbacks in this diagram involve only binary products. The crucial step involves the projection morphism 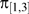, which removes the middle term 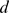, as it is no longer needed to obtain the inference via a second pullback. The key to this strategy is being able to segment the inference into a sequence of two steps.

A related situation also arises for Class Inclusion, which is shown in the following diagram
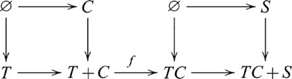
(36)where the morphism 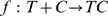 is not inclusion, but some recoding (chunking) of triangles and circles into instances of a common object type, TC. For example, a possible key to accessing this type of strategy is to recode on the basis of a common feature by asking the question *Are there more small shapes than shapes?* These two examples illustrate in category theory terms how the complexity of a task may be influenced by strategy.

## Discussion

Using category theory constructs, we have revealed a formal connection between Transitive Inference and Class Inclusion. Transitive Inference involves a categorical product of premise relations. Class Inclusion involves a coproduct between two complementary subclasses. In category theory, product and coproduct are dual. Thus, the formal connection between Transitive Inference and Class Inclusion is that they involve the “same” (isomorphic) processes in the categorical sense. This connection extends to other tasks establishing an equivalence class of inferential abilities formally based on the need to compute (co)products. In the simpler, one-dimensional version of Matrix Completion, the apparent product is isomorphic to a structure that does not involve a (co)product. Note that children are not required to first compute the (co)product to realize that it's reducible: they use a (co)product-free strategy which works, because of the simpler nature of the task. These results point to a fundamental principle under development during childhood that is the capacity to compute (co)products.

The implication that computing (co)products is fundamental to cognitive development raises two general questions: (1) Is the connection between these inferential abilities real, or just a coincidence? (2) If the connection is real, what does computing a (co)product mean in terms of possible neurocognitive processes? To the first question, as with any theory, one cannot rule out the possibility of being discounted by new data. The best one can hope for is to account for a wide variety of cases that are within the intended scope of the theory. In this regard, the empirical evidence now available and the variety of cases analyzed, both positive and negative conditions in each of seven paradigms, gives us cause for confidence that the connection is indeed real.

There are several caveats, however, in regard to establishing correspondences between paradigms and age groups. First, as already mentioned, an important consideration is the correlation across paradigms, not a specific age of achievement. Second, task knowledge and familiarity with materials will obviously be modulating factors. Third, in some cases, there may exist alternative task strategies that circumvent a particular level of complexity, as shown in the extended versions of Transitive Inference and Class Inclusion. These sorts of considerations have been discussed elsewhere in the context of Relational Complexity theory [32]. Hence, while we argue that categorical (co)product captures an important aspect of cognitive development, it is not intended to be the only consideration, nor is it necessarily incompatible with other approaches.

Category theory offers a potentially powerful approach to theorizing about cognition by not having to presuppose an, as yet, unknown internal structure for cognitive states representing task elements. Notice that the definition of a functor, and therefore duality (see Text S1) does not make reference to the elements within an object (i.e., an object's *internal* structure). The definitions refer only to the morphisms, which constrain the relationships between objects (i.e., their *external* structure). So, one is not required to make an *a priori* commitment to, say, symbolic or subsymbolic computational processes. In this sense, category theory complements more detailed (e.g., symbolic, or connectionist) approaches to cognitive modeling. In particular, we started with the difficulty that Transitive Inference and Class Inclusion poses for *Relational Complexity* and *Complexity and Cognitive Control* theories. From our categorical perspective, we now see that the Relational Complexity explanation of Transitive Inference (i.e., integration of two binary relations into a ternary relation) is a special case of a categorical product. The commutative diagrams for ternary (co)products also show how one may incorporate a levels of hierarchy explanation, as may be employed by Complexity and Cognitive Control theory, where a ternary (co)product may be computed from two binary (co)products.

While the abstractness afforded by category theory is generally seen as a strength, it leaves open the question of what exactly is being computed in these situations. To the second question, then, we look to neuroscience. One of the major attractions of category theory for mathematicians and computer scientists is that it offers abstraction (hence, generalization) *with* precision. Cognitive neuroscience research has implicated the prefrontal cortex as important for processing relational information [30], [33]–[35]. For example, patients with damage to prefrontal cortex were significantly worse on Transitive Inference and Class Inclusion tasks than normals and patients with anterior temporal cortical damage [35]. Adults, but not children (8–12 years old) showed sustained activity in rostrolateral prefrontal cortex during the more difficult two-relation than one-relation condition of a Raven's Progressive Matrices task [33]. The general suggestion has been that regions within the prefrontal cortex are responsible, in some informal sense, for the integration and maintenance of relational information [30]. Our category theory approach makes more precise claims in formal terms of pullbacks and pushouts.

Research on the neural basis of reasoning has focussed on localizing functionality to specific cortical regions, particularly within the prefrontal cortex. Yet, the commutative diagrams clearly show the importance of transformations between objects. One intriguing possibility is that the morphisms correspond to functional connectivity realized in part by long-distance cortical connections. An area where the neural basis of cognitive function has been studied in detail is visual attention (see [36]). Conjunctive visual search involves finding a target item among a display of non-targets, where the target is uniquely identified by a conjunction of features, such as colour and orientation. In categorical terms, conjunctive search involves a product of (e.g., colour and orientation) feature maps. Each feature map is a set of location-feature relationships, and their conjunction is the product of those maps constrained by location (i.e., a pullback). It is well-known that conjunctive search is more difficult (steeper search slope) than feature search (see [37]). Interestingly, a visual search study on monkeys using implanted electrodes revealed greater frontal-parietal neural synchrony in the lower gamma band (22–34 Hz) for conjunctive than feature search [38]. A corresponding significant increase in phase synchrony between frontal and parietal scalp electrodes in the same frequency band was also reported in humans [39]. Whether the product underlying conjunctive search relates to the products identified here remains to be determined. What this example illustrates is a further benefit of a categorical approach, where the methods of one field are shown to have novel applications in another—in this example, phase synchrony as an indicator of complexity.

A recurring theme in our analysis of these tasks is the integration (either multiplicatively, or additively) of multiple sources of information. Regions within the prefrontal cortex are often assigned this role, both anatomically and functionally (see [40] for a review). A general theory of intelligence proposes that maturation of the prefrontal cortex in coordination with other cortical regions is a key factor [41]. Hence, maturation of cortical connectivity is a possible biological basis for the observed correspondences in the development of inference, though we do not regard maturation as the only factor, as already discussed.

More generally, we have used category theory to propose new experiments that directly test comparisons and contrasts for all levels. The basis for determining whether tasks belong to the same level is isomorphism, either between objects or the diagrams (categories) to which they belong. In regard to the latter, we identified a subtle difference between diagrams containing constrained versus unconstrained (co)products. This difference speaks to the potential power of category theory in that it affords a finer grained analysis within the major levels defined by (co)product arity (i.e., unary, binary, ternary, etc). Although further work is needed to ascertain the empirical implications of these differences both within and across higher levels, the examples provided show how this work may proceed.

There are two main types of predictions for these extended paradigms that follow naturally from the arities of computed (co)oroducts. They are: (1) tasks involving (co)products of arity 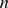 will yield significantly lower performance than tasks involving (co)products are arity 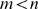 for participants within the same age group, excluding of course floor effects, where performance on neither task is above chance; and (2) tasks at the same arity will yield significance performance correlations. A corollary to these predictions is that older participants will generally outperform younger participants on a task at a given arity.

One may wonder whether other category theory-based models could account for the same developmental data. 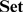 has been the categorical basis for our analysis. A natural alternative for modeling relations is the category 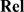, which has sets, 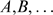, for objects; and for morphisms from 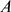 to 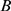, relations 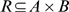, instead of functions, where the identity morphism on 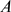 is the equality relation, 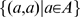; and composition defined so that for 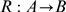 and 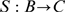, 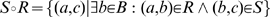. Composition in this category is essentially an equijoin. Thus, Transitive Inference on a set 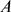 is represented more succinctly by a diagram consisting of a morphism, 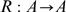 and its composition with itself, 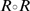, being the inference. However, Class Inclusion does not lend itself to a more succinct representation in 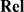, so the analogy between the two cannot be captured in 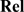. (The inclusion relations 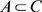 and 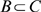 capture the data for Class Inclusion, but there is no valid composition operation in this context.) This example reinforces our earlier point that category theory, while abstract, is not an arbitrary fit-for-all formulation.

These two categorical bases for Transitive Inference (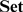 and 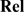) raise another point in regard to a notion of cognitive flexibility mentioned earlier. The 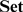 version of Transitive Inference indicates that to make the inference one must consider the constraining item 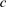 (in Diagram 14) from two perspectives conjointly: in the case of the blocks task, as the block that is both higher than 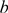 and lower than 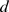. By contrast, in the 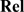 version, the inference relies on just one morphism or perspective, applied twice. This difference also has implications for developmental and comparative psychology in that simply demonstrating transitivity in infants and non-humans is not sufficient evidence of a cognitive capacity that is in some way equivalent to older children and adult humans. Our categorical (co)product formulation says that if they have the capacity for Transitive Inference in the same cognitively flexible manner, then they should also have the capacity for other inferences involving (co)products such as Class Inclusion, assuming a means of administering the test that is appropriate for the cohort.

Category theory affords a view of the forest despite the trees. It helps reveal unseen connections between (cognitive) structures. And, in doing so, the methods and results from one field become applicable to another. That was the original motivation for having a science of cognition.

## Supporting Information

Text S1 Duality
(0.09 MB PDF)
