# Supplementary material for: What Do Transitive Inference and Class Inclusion Have in Common? Categorical (Co)Products and Cognitive Development
Source: PLoS Comput Biol. 2009 Dec 11;5(12):e1000599. doi: 10.1371/journal.pcbi.1000599 (PMC2781167; doi:10.1371/journal.pcbi.1000599)
Supplement: Text S1 — Duality (0.09 MB PDF) [file pcbi.1000599.s001.pdf]

Duality

Duality in category theory is formally defined in terms of (isomorphic contravariant) functors. A *(covariant) functor* $F : \mathbf{C} \to \mathbf{D}$ is a structure-preserving morphism between categories $\mathbf{C}$ and $\mathbf{D}$ that associates each object A in $\mathbf{C}$ to an object $F(A)$ in $\mathbf{D}$; and each morphism $f : A \to B$ in $\mathbf{C}$ to a morphism $F(f) : F(A) \to F(B)$ in $\mathbf{D}$, such that $F(1_A) = 1_{F(A)}$ for each object A in $\mathbf{C}$; and $F(g \circ_{\mathbf{C}} f) = F(g) \circ_{\mathbf{D}} F(f)$ for all morphisms $f : A \to B$ and $g : B \to C$ for which compositions $\circ_{\mathbf{C}}$ and $\circ_{\mathbf{D}}$ are defined in categories $\mathbf{C}$ and $\mathbf{D}$, respectively. A *contravariant functor* $\overline{F} : \mathbf{C^{op}} \to \mathbf{D}$ is a morphism $\overline{F}(\overline{f}) : \overline{F}(\overline{B}) \to \overline{F}(\overline{A})$ that preserves identities and compositions, such that $\overline{F}(\overline{f} \circ_{\mathbf{C^{op}}} \overline{g}) = \overline{F}(\overline{g}) \circ_{\mathbf{D}} \overline{F}(\overline{f})$. A functor $F : \mathbf{C} \to \mathbf{D}$ is an *isomorphic functor*, if and only if there exists a functor $G : \mathbf{D} \to \mathbf{C}$ such that $G \circ F = 1_{\mathbf{C}}$ and $F \circ G = 1_{\mathbf{D}}$, where $1_{\mathbf{C}}$ and $1_{\mathbf{D}}$ are the identity functors sending objects and morphisms to themselves. Two categories, or constructs are *dual* if there exists an isomorphic contravariant functor transforming one to the other.

It is straightforward to show that there exists an isomorphic contravariant functor between product and coproduct. Here, we rename ambiguous labels in the category $\mathbf{Q}$ containing the coproduct. First, suppose a morphism $\overline{F} : \mathbf{Q} \to \mathbf{P}$, such that $\overline{F}(Q) = P$, $\overline{F}(q_i) = p_i$, and $\overline{F}(\overline{A}) = A$, etc. Assuming identity morphisms, then $\overline{F}$ is a functor, since $\overline{F}(\overline{u} \circ_{\mathbf{Q}} q_1) = \overline{F}(\overline{z_1}) = z_1 = p_1 \circ_{\mathbf{P}} u = \overline{F}(q_1) \circ_{\mathbf{P}} \overline{F}(\overline{u})$, and similar derivations apply to the other compositions. Since there exists a functor $F : P \to Q$, which is simply the reverse of $\overline{F}$ (i.e., $\overline{F} \circ F(A) = A$, and $\overline{F} \circ F(p_1) = p_1$, etc), then $\overline{F} \circ F = 1_{\mathbf{P}}$ and $F \circ \overline{F} = 1_{\mathbf{Q}}$. So, $\mathbf{P} \cong \mathbf{Q}$ (isomorphic), hence product and coproduct are dual.
